# 

**Published:** 1907-03

**Authors:** 


### CONTENTS.

ORIGINAL COMMUNICATIONS—
The Clinical Significance of Blood Examinations in Reference to Surgical Diagnosis and Prognosis. By Willis Jones, M. D., Atlanta Ga
Foreign Bodies in the Facial Orifices. By Richard R. Daly, M. D., Atlanta, Ga
A Combination Operating Fracture and Orthapædic Table. By J. H. Downey, M. D., Gainesville, Ga
Discussion of Dr. Downey's Paper (Informal)
EDITORIAL NOTES AND COMMENTS—
Uncinariasis
Exophthalmic Goitre
To the Medical Profession of Georgia, Florida and South Carolina
Anti-Tuberculosis Agitation
BOOK REVIEWS
SELECTIONS AND ABSTRACTS—
Castration Instead of Lynching
Home and Office Treatment of Inebriety
Value of the Leucocyte Count
The Oatmeal Cure in Diabetes
A Review of Over Fourteen Thousand Surgical Anæthesias
Surgical Shock
The Practice of Medicine
Milk and Tuberculosis Disease
The Diagnosis Between Duodenal Ulcer and Gall Stone Disease
A Further Study of Perforation of the Bowel in Typhoid Fever
The Physician in Literatnre
Symptoms of Cancer of the Uterus
Chloridæmia in Bright's Disease
Goitre
Sulphate of Spartein in Surgical Practice. By Stuart McGuire, M. D., Surgeon in Charge St. Luke's Hospital Richmond, Va
Strontium Bromide in Epilepsy
Some Points in the Diagnosis and Treatment of Accidental Hemorrhage
The Oral Administration of Antitoxin
Osteopathic Legislation for District of Columbia
The Basis and Application of Wright's Opsonin Theory. By Dr E Weinstein
Coujunctivitis
Danger of Scopolamin-Morphin Anesthesia

